# Comparing the efficacy of different methods in assessing cervical stromal invasion in endometrial carcinoma: a retrospective study of 2,020 patients

**DOI:** 10.3389/fonc.2025.1548436

**Published:** 2025-02-11

**Authors:** Ying Yang, Zhijun Ye, Yifei Zhao, Zhengyu Li

**Affiliations:** ^1^ Department of Gynecology and Obstetrics, West China Second University Hospital, Sichuan University, Chengdu, China; ^2^ Key Laboratory of Birth Defects and Related Diseases of Women and Children (Sichuan University), Ministry of Education, Chengdu, China; ^3^ Department of Radiology, West China Second University Hospital, Sichuan University, Chengdu, China

**Keywords:** endometrial carcinoma, cervical stromal invasion, MRI, CT, endometrial biopsy, diagnosis

## Abstract

**Purpose:**

This study aimed to assess the preoperative diagnostic efficacy of magnetic resonance imaging (MRI), computed tomography (CT), and endometrial biopsy for cervical stromal invasion (CSI) in endometrial carcinoma (EC) and to discuss the influencing factors of CSI.

**Material and methods:**

A total of 2,020 patients with EC were retrospectively analyzed in a tertiary hospital. Basic patient information, clinical pathology, and laboratory indicators were collected and analyzed. Using the postoperative pathological diagnosis as the gold standard, the diagnostic efficacies of different preoperative methods were analyzed. Additionally, influencing factors of CSI were examined by univariate and multivariate analyses.

**Results:**

The sensitivity (Sens.), specificity (Spec.), accuracy (Acc.), diagnostic odds ratio (DOR), Youden’s index, and Kappa value of the MRI vs. CT groups were 49.50% vs. 56.74%, 92.24% vs. 79.09%, 87.70% vs. 76.15%, 11.60 vs. 4.93, 0.42 vs. 0.36, and 0.392 vs. 0.256 (*p* < 0.001), respectively. The Sens., Spec., Acc., DOR, Youden’s index, and Kappa value of the endometrial biopsy group were 41.74%, 93.25%, 87.08%, 9.97, 0.35, and 0.363 (*p* < 0.001), respectively. CSI was associated with cancer antigen 125, myometrial invasion, adnexal invasion, parametrial invasion, lymph node metastasis, and progesterone receptor.

**Conclusions:**

MRI is relatively superior in assessing CSI, although diagnostic authenticity and consistency were unsatisfactory. Combining MRI and biopsy could improve diagnostic sensitivity, aiding in clinical decision making and prognostic prediction. Comprehensive consideration of high-risk factors for the occurrence of CSI may aid the diagnosis. Preoperative diagnostic methods of CSI in EC still need to be explored further to improve efficiency.

## 1 Introduction

Endometrial carcinoma (EC) is the fourth most common female malignant tumor in the world. In 2002, 198,783 new cases of EC were recorded, which increased to 417,367 in 2020. EC-related deaths worldwide tallied 50,327 in 2002 and increased to 97,370 in 2020 (1–3). Cervical stromal invasion (CSI) is classified as stage IIA by the International Federation of Gynecology and Obstetrics 2023 and accounts for approximately 12% of EC cases. Accurate diagnosis of CSI is needed to guide the surgery and postoperative adjuvant treatment protocol and predict the prognosis and survival outcome of patients. The National Comprehensive Cancer Network guidelines indicate that patients with CSI should be treated via total or radical hysterectomy plus bilateral salpingo-oophorectomy and surgical staging, especially when a negative margin is required (4). CSI is a high-risk factor for supplementary radiotherapy and chemotherapy and is an independent predictor of death; the 5-year overall survival of patients with CSI ranges from 72.9% to 85.8% (5–8). Moreover, the exclusion of CSI is necessary for young patients with the chance of receiving fertility-preserving surgery.

Currently, the general methods used to diagnose CSI preoperatively include endometrial biopsy, magnetic resonance imaging (MRI), and computed tomography (CT). Endometrial biopsy can clarify the histological classification and grading of tumors and molecular classification, guiding prognostic risk stratification and treatment decisions (9–11). Nicole et al. (2017) demonstrated a diagnostic accuracy of 60%–79% for EC with traditional dilatation and curettage and 80%–98% with hysteroscopy, but a lower diagnostic efficacy for CSI (12–14). MRI is considered superior for assessing the depth of myometrial invasion, the condition of the endometrium, and lymph node (LN) status (15). The interpretation of MRI depends on differences in signal intensity across tissues; misdiagnosis often occurs when endometrial and endocervical canals are distended or the invasion of cervical stroma proceeds from the adjacent myometrium. The reported diagnostic sensitivity (Sens.), specificity (Spec.), and accuracy (Acc.) of CSI by MRI ranges from 33% to 69%, 82% to 96%, and 46% to 89%, respectively (16–18); however, the diagnostic efficiency varied across studies and with different sequences. CT is commonly used to assess tumor size, extent, blood supply, and distant metastasis, which is considered with low resolution for soft tissue imaging. Hardesty et al. (2001) showed that CT Sens. and Spec. were 83% and 42%, respectively, in evaluating CSI (19). The best diagnostic method for CSI remains unspecified. Therefore, this study aimed to explore the optimal method for diagnosing CSI in EC preoperatively, which may lead to more personalized clinical treatment.

## 2 Materials and methods

### 2.1 Study design and participants

This study was approved by the Medical Ethics Committee, and all the enrolled patients have signed informed consent forms. Data from EC cases were retrospectively analyzed in a tertiary hospital from January 2017 to January 2022. Basic patient information, including clinical pathological and laboratory detection indicators, was extracted through the Hospital Information System and jointly completed by three trained doctors.

Inclusion criteria were as follows: 1) diagnosis of EC based on either preoperative endometrial biopsy results or postoperative pathological results; 2) diagnostic methods contained preoperative endometrial biopsy and at least one imaging examination, and biopsy samples or imaging from external hospitals was examined by our own professional doctors with >5 years of work experience; 3) patients underwent standardized EC staging surgery (including hysterectomy, bilateral salpingo-oophorectomy, and sentinel lymph node mapping or systematic lymphadenectomy (20)), and the specimens were dissected and sent for pathological examination. Patients were excluded if they met any of the following criteria: 1) underwent fertility-preserving treatment at least once, 2) received preoperative neoadjuvant chemotherapy, or 3) displayed other malignant tumors of the uterus, congenital or acquired immune deficiency, or severe heart, lung, liver, and/or kidney dysfunction.

CSI was assessed according to the European Society of Urogenital Radiology guidelines (15). CSI was considered when hypointense cervical stroma was disrupted by hyperintense tumor region, or low signal intensity of normal cervical loop was incomplete or irregular on T2-weighted imaging acquired on a 3-T scanner (Siemens Healthcare, Erlangen, Germany). At least two expert radiologists (more than 5 years’ experience) worked in consensus to outline the diagnostic reports. CSI was indicated when a low-density tumor or heterogeneous enhancement appeared in cervical stroma or the neoplastic lesions of the endometrium extended into cervical stroma on CT (21). CSI was diagnosed when cervical tissue showed cytologic features of neoplastic cells such as microscopic morphological variants, lack of border, and lack of polarity on endometrial biopsy pathology.

### 2.2 Statistical analysis

Data were analyzed using SPSS 26.0 (IBM Corp., Armonk, NY, USA); missing data were completed by multiple imputation. Normality of the data was tested using the Kolmogorov–Smirnov normality test. When the quantitative data satisfied normality, the mean and standard deviation were used and analyzed by two-independent samples *t*-test or analysis of variance for two or more samples, respectively. When the quantitative data did not satisfy normality, the median and interquartile were used to describe the data and were analyzed using the Mann–Whitney *U* test or Kruskal–Wallis *H* test for two or more samples, respectively. Qualitative data were statistically described as constituent ratios, and the differences between groups were analyzed by chi-square test. Additionally, the exploration of influencing factors was conducted by univariate analysis and binary logistic regression analysis.

Considering the postoperative pathological diagnosis as the gold standard, we used the following indicators to evaluate the effectiveness of the diagnostic methods: Sens., Spec., Acc., false positive rate (FPR), false negative rate (FNR), positive predictive value (PPV), negative predictive value (NPV), positive likelihood ratio (LR+), negative likelihood ratio (LR−), diagnostic odds ratio (DOR), Youden’s index, and Kappa value. Acc. indicates the percentage of correctly diagnosed patients (true positive and true negative) among all patients. PPV refers to the true ratio of positive results obtained by a specific test, while NPV refers to the true ratio of negative results. Additionally, when LR+ > 10 or LR− < 0.1, the diagnosis or exclusion of specific diseases was considered more credible. Furthermore, when Youden’s index and Kappa test values were more similar, diagnostic authenticity and consistency improved.

## 3 Results

### 3.1 Study population

**Figure 1:**
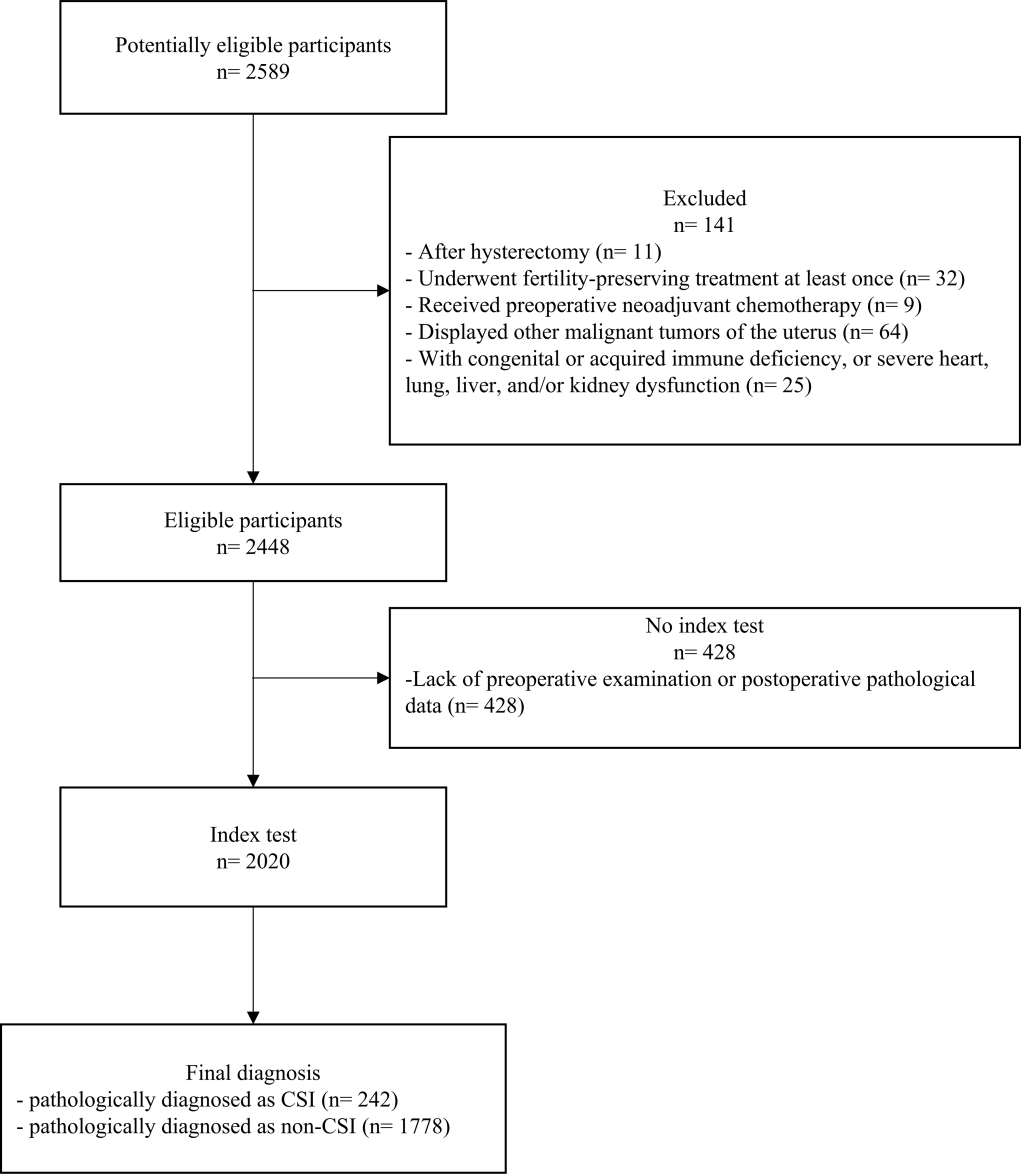
Flowchart of the inclusion and exclusion criteria of participants with endometrial carcinoma with or without cervical stromal invasion. CSI, cervical stromal invasion.

A total of 2,020 patients were enrolled in this study and grouped by diagnosis: 242 cases were pathologically diagnosed as CSI and 1,778 cases were non-CSI (**Figure 1**, **Table 1**). The incidence of CSI was 12.0%, which was in accordance with previous reports of 5%–12% (22). The median age of patients was 53 years in the CSI group and 52 years in the non-CSI group. The number of patients with diabetes was 20 (8.3%) in the CSI group and 165 (9.3%) in the non-CSI group. There were no significant differences in age, blood type, or diabetes between the CSI and non-CSI groups. The median value of cancer antigen 125 (CA125) was 31.55 U/mL (interquartile = 73.8) in the CSI group and 13.40 U/mL (interquartile = 24.2) in the non-CSI group (*p* < 0.001). The incidence of obesity (87.6% vs. 81.5%), non-endometrioid histological type (41.7% vs. 13.9%), and advanced histological grading (29.8% vs. 9.3%) were more frequent in the CSI group than in the non-CSI group (*p* < 0.05). The incidence of positive peritoneal cytology (9.1% vs. 3.3%), deep myometrial invasion (56.2% vs. 17.8%), lymphovascular space invasion (LVSI) (50.0% vs. 15.5%), adnexal invasion (31.8% vs. 8.3%), parametrial invasion (19.0% vs. 1.7%), and LN metastasis (34.3% vs. 4.7%) were higher in the CSI group than in the non-CSI group (*p* < 0.001). The rates of positive estrogen receptor and progesterone receptor detection by immunohistochemistry were lower in the CSI group than in the non-CSI group (74.4% vs. 92.9%, *p* < 0.001; 58.7% vs. 87.3%, *p* < 0.001), whereas the rates of abnormal p53 status, Ki67 overexpression, and deficient mismatch repair (dMMR) status were higher in the CSI group than in the non-CSI group (all *p* < 0.05).

**Table 1:** General characteristics of patients with or without cervical stromal invasion.

	CSI (n = 242, %)	Non-CSI (n = 1,778, %)	Chi-square value	*p*-Value
Age (years) [M (Q)]	53 (11)	52 (11)		0.113
Blood type			3.042	0.385
A	70 (28.9)	583 (32.8)		
B	67 (27.7)	453 (25.5)		
O	75 (31.0)	570 (32.1)		
AB	30 (12.4)	172 (9.7)		
History of diabetes	20 (8.3)	165 (9.3)	0.264	0.607
Obesity	212 (87.6)	1,449 (81.5)	5.437	**0.020**
Serum CA125 level (U/mL) [M (Q)]	31.55 (73.8)	13.40 (24.2)		**<0.001**
Positive peritoneal cytology	22 (9.1)	59 (3.3)	18.441	**<0.001**
Histological subtype			126.111	<0.001
Precursors of carcinoma	0	140 (7.9)		
Endometrioid carcinoma	141 (58.3)	1,391 (78.2)		
Non-endometrioid carcinoma	101 (41.7)	247 (13.9)		
Serous	11 (10.9)	49 (19.8)		
Clear cell	9 (8.9)	20 (8.1)		
Carcinosarcoma	9 (8.9)	25 (10.1)		
Mixed cell	44 (43.6)	81 (32.8)		
Undifferentiated/ Dedifferentiated	18 (17.8)	15 (6.1)		
Others	10 (9.9)	57 (23.1)		
Histological grade			78.217	<0.001
G1	47 (33.3)	923 (66.4)		
G2	52 (36.9)	339 (24.4)		
G3	42 (29.8)	129 (9.3)		
Myometrial invasion			180.260	<0.001
<50%	106 (43.8)	1,461 (82.2)		
≥50%	136 (56.2)	317 (17.8)		
LVSI	121 (50.0)	275 (15.5)	161.172	**<0.001**
Adnexal invasion	77 (31.8)	148 (8.3)	118.788	**<0.001**
Parametrial invasion	46 (19.0)	30 (1.7)	176.495	**<0.001**
LN metastasis	83 (34.3)	83 (4.7)	247.928	**<0.001**
IHC				
ER+	174 (74.4)	1,457 (92.9)	81.680	**<0.001**
PR+	138 (58.7)	1,366 (87.3)	121.300	**<0.001**
Abnormal p53 status	111 (52.6)	669 (43.2)	6.593	**0.010**
Ki67 overexpression	174 (73.7)	842 (54.1)	32.226	**<0.001**
dMMR	60 (33.5)	329 (24.0)	7.541	**0.006**

CSI, cervical stromal invasion; LVSI, lymphovascular space invasion; LN, lymph node; IHC, immunohistochemistry; ER, estrogen receptor; PR, progesterone receptor; dMMR, deficient mismatch repair. Results with statistical significance (p < 0.05) are shown in bold.

### 3.2 Diagnostic efficacy of different methods

Patients were grouped by diagnostic method; there were 951 (47.1%) patients in the MRI group and 1,069 (52.9%) patients in the CT group. All patients had preoperative endometrial biopsy results and postoperative pathological diagnosis results. Baseline features between the two groups were basically consistent (**Table 2**).

**Table 2:** Baseline features of patients in MRI and CT groups.

	MRI (n = 951, %)	CT (n = 1,069, %)	Chi-square value	*p*-Value
Age (years) [M (Q)]	52 (10)	53 (11)		**0.003**
Obesity	777 (81.7)	884 (82.7)	0.338	0.561
History of diabetes	97 (10.2)	88 (8.2)	2.342	0.126
Serum CA125 level (U/mL) [M (Q)]	13.10 (21.1)	15.50 (40.6)		**<0.001**
Positive peritoneal cytology	33 (3.5)	48 (4.5)	1.361	0.243
Histological subtype			9.09	0.011
Precursors of carcinoma	79 (8.3)	61 (5.7)		
Endometrioid carcinoma	727 (76.4)	805 (75.3)		
Non-endometrioid carcinoma	145 (15.3)	203 (19.0)		
Serous	29 (20.0)	31 (15.3)		
Clear cell	12 (8.3)	17 (8.4)		
Carcinosarcoma	19 (13.1)	15 (7.4)		
Mixed cell	47 (32.4)	78 (38.4)		
Undifferentiated/ Dedifferentiated	9 (6.2)	24 (11.8)		
Others	29 (20.0)	38 (18.7)		
Histological grade			4.416	0.110
G1	480 (66.0)	490 (60.9)		
G2	173 (23.8)	218 (27.1)		
G3	74 (10.2)	97 (12.0)		
Myometrial invasion			3.023	0.082
<50%	754 (79.3)	813 (76.1)		
≥50%	197 (20.7)	256 (23.9)		
LVSI	170 (17.9)	226 (21.1)	3.405	0.065
Adnexal invasion	90 (9.5)	135 (12.6)	5.039	**0.024**
Parametrial invasion	31 (3.3)	45 (4.2)	1.254	0.263
LN metastasis	75 (7.9)	91 (8.5)	0.262	0.609

MRI, magnetic resonance imaging; CT, magnetic resonance imaging; LVSI, lymphovascular space invasion; LN, lymph node. Results with statistical significance (p < 0.05) are shown in bold.

The evaluation of different diagnostic methods compared with the gold standard of postoperative pathological diagnosis is shown in **Table 3**. The Sens., Spec., Acc., FPR, and FNR of the MRI group were 49.50%, 92.24%, 87.70%, 7.76%, and 50.50%, respectively. The PPV was 43.10% and NPV was 93.89%, indicating that 93.89% of patients were diagnosed as non-CSI by pathology after surgery, among patients who were diagnosed as non-CSI by MRI before surgery. The LR+, LR−, and DOR were 6.38, 0.55, and 11.60, respectively. Youden’s index indicates the ability of diagnostic methods to identify people with or without CSI. The larger the index, the higher the authenticity. In this study, Youden’s index for the MRI group was 0.42. Kappa values closer to 1 indicated greater consistency. In the MRI group, the Kappa value was 0.392 (*p* < 0.001), indicating poor consistency.

**Table 3:** Diagnostic values of different methods for cervical stromal invasion in endometrial carcinoma.

Diagnostic methods	Sens. (%)	Spec. (%)	Acc. (%)	FPR (%)	FNR (%)	PPV (%)	NPV (%)	LR+	LR−	DOR	Youden’s index	Kappa value (*p*-Value)
Image examinations												
MRI	49.50	92.24	87.70	7.76	50.50	43.10	93.89	6.38	0.55	11.60	0.42	**0.392 (*p* < 0.001)**
CT	56.74	79.09	76.15	20.91	43.26	29.20	92.33	2.71	0.55	4.93	0.36	**0.256 (*p* < 0.001)**
**Endometrial biopsy**	41.74	93.25	87.08	6.75	58.26	45.70	92.16	6.18	0.62	9.97	0.35	**0.363 (*p* < 0.001)**
Combined methods												
MRI and biopsy	60.40	86.12	83.39	13.88	39.60	34.08	94.82	4.35	0.46	9.46	0.47	**0.347 (*p* < 0.001)**
CT and biopsy	68.79	74.89	74.09	25.11	31.21	29.39	94.05	2.74	0.42	6.52	0.44	**0.279 (*p* < 0.001)**

MRI, magnetic resonance imaging; CT, magnetic resonance imaging; Sens., sensitivity; Spec., specificity; Acc., accuracy; FPR, false positive rate; FNR, false negative rate; PPV, positive predictive value; NPV, negative predictive value; LR+, positive likelihood ratio; LR−, negative likelihood ratio; DOR, diagnostic odds ratio. Results with statistical significance (p < 0.05) are shown in bold.

The Sens., Spec., Acc., FPR, FNR, PPV, NPV, LR+, LR−, and DOR of the CT group were 56.74%, 79.09%, 76.15%, 20.91%, 43.26%, 29.20%, 92.33%, 2.71, 0.55, and 4.93, respectively. Youden’s index was 0.36, and the Kappa value was 0.256 (*p* < 0.001), indicating poor consistency.

The Sens., Spec., Acc., FPR, FNR, PPV, NPV, LR+, LR−, and DOR of the endometrial biopsy group were 41.74%, 93.25%, 87.08%, 6.75%, 58.26%, 45.70%, 92.16%, 6.18, 0.62, and 9.97, respectively. Youden’s index was 0.35, and the Kappa value was 0.363 (*p* < 0.001), indicating poor consistency.

CSI was considered in groups of MRI or CT combined with endometrial biopsy. The Sens., Spec., Acc., FPR, FNR, PPV, NPV, LR+, LR−, and DOR of the MRI combined with the endometrial biopsy group were 60.40%, 86.12%, 83.39%, 13.88%, 39.60%, 34.08%, 94.82%, 4.35, 0.46, and 9.46, respectively. Youden’s index was 0.47, and the Kappa value was 0.347 (*p* < 0.001), indicating poor consistency. The Sens., Spec., Acc., FPR, FNR, PPV, NPV, LR+, LR−, and DOR of the CT combined with the endometrial biopsy group were 68.79%, 74.89%, 74.09%, 25.11%, 31.21%, 29.39%, 94.05%, 2.74, 0.42, and 6.52, respectively. Youden’s index was 0.44, and the Kappa value was 0.279 (*p* < 0.001), indicating poor consistency.

### 3.3 Factors affecting the diagnostic efficiency of MRI

As the diagnostic efficacy of MRI was relatively superior but still unsatisfactory, we mainly analyzed influencing factors to explore possible ways to improve efficacy. We analyzed 101 cases in the MRI group with postoperatively diagnosed CSI: 50 of the postoperative pathological diagnoses were consistent with preoperative diagnosis, whereas 51 cases were inconsistent. Univariate analysis indicated that there were no significant differences in age, obesity, histological type and grade, myometrial invasion, LVSI, adnexal invasion, parametrial invasion, LN metastasis, or the invasion depth of stroma (*p* > 0.05). Invasion depth categorization into superficial or deep CSI groups did not alter significance regardless of the depth threshold used (>1/2 or >2/3).

To explore the impact of radiologists’ subjective judgment on the diagnosis of CSI, 73 cases where preoperative reports were inconsistent with the postoperative pathological diagnosis were reassessed by another well-experienced radiologist. Compared with the gold standard of postoperative pathological diagnosis, the Sens., Spec., and Acc. of reassessment data in the diagnosis of CSI were 48.57%, 89.47%, and 69.86%, respectively. Reassessing the MRI diagnosis did not improve the diagnostic efficiency, indicating certain limitations of MRI in the diagnosis of CSI.

### 3.4 Main factors affecting CSI

The results of the bivariate logistic multivariate regression analysis showed that CSI was associated with CA125 [odds ratio (OR): 2.031, 95% CI: 1.379–2.993, *p* < 0.001], myometrial invasion (OR: 2.599, 95% CI: 1.695–3.985, *p* < 0.001), adnexal invasion (OR: 1.829, 95% CI: 1.111–3.014, *p* = 0.018), parametrial invasion (OR: 2.602, 95% CI: 1.177–5.753, *p* = 0.018), LN metastasis (OR: 2.108, 95% CI: 1.237–3.592, *p* = 0.006), and progesterone receptor (OR: 0.534, 95% CI: 0.290–0.981, *p* = 0.043). However, the groups did not differ significantly in terms of age, obesity, peritoneal cytology, histological subtype, LVSI, estrogen receptor, p53, Ki67, or dMMR status (**Table 4**).

**Table 4:** Main factors affecting cervical stromal invasion according to bivariate logistic multivariate regression analysis.

	B value	Wald chi-square	OR (95% CI)	*p*-Value
Age	−0.007	0.464	0.993 (0.973–1.013)	0.496
Obesity	−0.029	0.014	0.971 (0.596–1.583)	0.906
Positive CA125	0.709	12.849	2.031 (1.379–2.993)	**<0.001**
Positive peritoneal cytology	0.482	1.744	1.619 (0.792–3.308)	0.187
Endometrioid carcinoma	−0.438	2.712	0.645 (0.363–1.087)	0.100
Deep myometrial invasion	0.955	19.174	2.599 (1.695–3.985)	**<0.001**
LVSI	0.250	1.151	1.284 (0.813–2.026)	0.283
Adnexal invasion	0.604	5.625	1.829 (1.111–3.014)	**0.018**
Parametrial invasion	0.956	5.578	2.602 (1.177–5.753)	**0.018**
LN metastasis	0.746	7.510	2.108 (1.237–3.592)	**0.006**
IHC				
ER+	−0.046	0.015	0.955 (0.461–1.979)	0.901
PR+	−0.628	4.092	0.534 (0.290–0.981)	**0.043**
Abnormal p53 status	−0.335	2.919	0.715 (0.487–1.051)	0.088
Ki67 overexpression	0.168	0.635	1.183 (0.782–1.791)	0.425
dMMR	0.362	3.088	1.436 (0.969–2.149)	0.079

LVSI, lymphovascular space invasion; LN, lymph node; IHC, immunohistochemistry; ER, estrogen receptor; PR, progesterone receptor; dMMR, deficient mismatch repair; OR, odds ratio; 95% CI, confidence interval. Results with statistical significance (p < 0.05) are shown in bold.

## 4 Discussion

The incidence rate of EC ranks first among malignant tumors of the female reproductive system in developed countries and ranks second in China, severely threatening women’s health. The diagnosis of CSI in EC is of great significance for assessing the International Federation of Gynecology and Obstetrics stage, surgical and postoperative adjuvant protocol, and prognosis. Clinical guidelines have recommended treating patients with CSI with simple, radical, or modified radical hysterectomy; although the differences in 5-year overall survival in different procedures were disputed, radical or modified radical hysterectomy were essential when negative margins were needed (23, 24). Moreover, external radiation therapy was recommended as the first choice for EC patients with CSI, which can be combined with intracavitary radiation and systemic therapy based on personal conditions. The scope of radiation therapy included the lymphatic drainage areas of the common, internal and external iliac, obturator, and presacral lymph nodes, as well as the upper vaginal, parametrial, and paravaginal segments (25). However, no research has specified whether a different surgery or lymphadenectomy would affect the efficacy of adjuvant radiotherapy and chemotherapy in patients with CSI.

The optimal preoperative diagnostic method for CSI is controversial. A meta-analysis including 14 studies performed by Bi et al. (2020) indicated that the diagnostic Sens., Spec., LR+, LR−, and DOR of MRI in CSI were 0.53, 0.95, 10.99, 0.46, and 29.50 (26), respectively, which were similar to our study findings. The invasion depth of cervical stroma >2/3 usually showed poor prognosis; nevertheless, no statistically significant differences were observed between CSI groups with >1/2 compared to >2/3 or <50% compared to >50% cervical stroma invasion depth (27), and the diagnostic efficacy did not improve after reassessment by another professional radiologist, indicating that MRI-related sequences had specific limitations in identifying CSI. Other MRI combined sequences or radiomics may solve this problem: several studies found that T2-weighted imaging combined with dynamic contrast-enhanced or diffusion-weighted imaging could improve diagnostic efficiency (28–30). Compared to traditional diagnostics, radiomics provides more quantitative and objective information. Recent studies have demonstrated that MRI-based radiomics models could predict CSI with higher accuracy than radiologists (0.972 vs. 0.711, *p* < 0.05) and improve precision diagnosis and treatment (31, 32).

Furthermore, when MRI was combined with endometrial biopsy, the diagnostic Sens. increased to 60.40% and the NPV increased to 94.82%. The reason for the high Spec. and low Sens. of MRI may be that macroscopic CSI is easily recognized, but microscopic CSI may be ignored; the low consistency of the combined methods may be explained by the fact that our study defined positive results as either test being positive.

Most studies of endometrial biopsy have focused on the differences between endometrial sampling methods for the diagnosis of EC, as well as the ability to recognize pathological types and grades. Our study did not distinguish between specific sampling methods because the samples of certain patients were re-analyzed at other hospitals. A meta-analysis based on 45 studies including 12,459 cases indicated that the diagnostic consistency rate of EC between hysteroscopy and postoperative pathology was 0.89, while the consistency rate between traditional curettage and postoperative pathology was 0.70; this was the lowest in the G2 group (33). In this study, the diagnostic Sens. of preoperative endometrial biopsy of CSI was 41.60% and the Spec. was 93.22%. The high Spec. and the low Sens. were potentially due to the reassessment of specimens from other hospitals. Some researchers have recommended hysteroscopy most in preoperative biopsies, but whether the use of uterine distending medium increases the risk of EC metastasis and the risk of positive peritoneal cytology requires more evidence to elucidate.

CSI is associated with other risk factors for poor prognosis (34), such as histological type, deep muscular invasion, LVSI, LN metastasis, and ovarian invasion (35, 36). In this study, when patients displayed high CA125, deep myometrial invasion, adnexal invasion, parametrial invasion, or LN metastasis, the probability of CSI increased, which is consistent with previous studies (37, 38). Positive peritoneal cytology was often considered a risk factor for EC; however, in this study, it was not associated with CSI. When cervical stroma was involved in EC, the LN metastasis pathway was considered similar to that of cervical cancer, which is among the reasons for poor prognosis. In addition, multiple immunohistochemical results related to molecular classification were also included in this study. Multivariate analysis showed that CSI was related to progesterone receptor, but not to the expression levels of estrogen receptor, p53, Ki67, or dMMR. Positive progesterone receptor was a protective factor for CSI. The association between CSI and molecular classification remains largely unknown, recent studies have highlighted the importance of combining morphological and molecular features in predicting prognosis (39), and further research is needed to understand the correlation.

A major advantage of this study was the large sample size, which provided a foundation for persuasive analysis. Moreover, we incorporated the latest immunohistochemical results related to molecular classification, closely combining cutting-edge research and clinical practice to obtain comprehensive results. The main limitation of this study was that its retrospective design may have resulted in selection and information biases in the dataset. Comparisons of different methods of preoperative endometrial biopsy and differences between MRI sequences or radiomics in the diagnosis of CSI require further exploration.

## 5 Conclusions

MRI is relatively superior in assessing cervical CSI, with higher Spec., Acc., and DOR among the preoperative diagnostic methods of CSI in EC. The combination of MRI and endometrial biopsy improves the diagnostic Sens. and NPV, despite unsatisfactory authenticity and consistency. Considering the influencing factors such as positive CA125, deep myometrial invasion, adnexal or parametrial invasion, lymph node metastasis, and negative progesterone receptor status, potentially enhances preoperative diagnosis. Furthermore, it would be meaningful to implement radiologist training programs and encourage the application of emerging radiomics based on machine learning and artificial intelligence. Preoperative diagnostic methods of CSI in EC require further research to improve efficiency.

## Data Availability

The raw data supporting the conclusions of this article will be made available by the authors, without undue reservation.
